# Green-Synthesized Magnesium Hydroxide Nanoparticles Induced Osteoblastic Differentiation in Bone Co-Cultured Cells

**DOI:** 10.3390/ph14121281

**Published:** 2021-12-08

**Authors:** Laura Costa Pinho, Marta M. Alves, Bruno Colaço, Maria Helena Fernandes, Catarina Santos

**Affiliations:** 1 Department of Animal Science, University of Trás-os-Montes and Alto Douro, 5000-801 Vila Real, Portugal; bcolaco@utad.pt; 2 Laboratory for Bone Metabolism and Regeneration, Faculty of Dental Medicine, University of Porto, 4200-393 Porto, Portugal; mhfernandes@fmd.up.pt; 3 CQE Instituto Superior Técnico, Universidade de Lisboa, 1049-001 Lisboa, Portugal; martamalves@tecnico.ulisboa.pt; 4 LAQV/Requimte, University of Porto, 4100-007 Porto, Portugal; 5 CECAV—Animal and Veterinary Research Centre UTAD, University of Trás-os-Montes and Alto Douro, 5000-801 Vila Real, Portugal; 6 EST Setúbal, CDP2T, Instituto Politécnico de Setúbal, Campus IPS, 2910-761 Setúbal, Portugal

**Keywords:** green synthesis, Mg(OH)_2_ nanoparticles, rose hip extract, co-cultured bone cells, osteoblastic induction

## Abstract

In this work, magnesium hydroxide NPs were synthesized using water (Mg(OH)_2_ NPs) or a rose hip (RH) extract (Mg(OH)_2_RH NPs) and tested for the bone cells’ effects in co-cultured osteoblastic and osteoclastic cells, using a Transwell^®^ insert system, allowing reciprocal cell paracrine interactions. Behavior of each cell population was characterized for typical phenotype markers, at days 1 and 6. Cell cultures treated with osteogenic/osteoclastogenic inducers were used as positive control of cell differentiation. The NPs presented a round shape morphology with an average diameter ~90 nm (Mg(OH)_2_ NPs) and below 10 nm (Mg(OH)_2_RH NPs. Both NPs induced osteoblastic and osteoclastic behavior similarly to that observed in induced osteoblastic and osteoclastic cultures (positive controls). Differences between the two types of particles were evident at the gene expression level. Compared to Mg(OH)_2_ NPs, the green-synthesized NPs greatly increased the expression of osteoblastic genes coding for the early markers ALP and collagen type 1 and the later transcription factor osterix, while decreasing the expression of osteoclastogenic genes, namely the essential transcription factor NFATC1, TRAP and the genes coding for the functional markers CA2 and CTSK. Overall, a positive added effect could be hypothesized for Mg(OH)_2_RH NPs with potential usefulness to promote bone formation in regenerative applications.

## 1. Introduction

Bone remodeling, occurring continuously in the bone microenvironment, is a process that comprises an equilibrium between bone resorption by the osteoclasts and bone formation by the osteoblasts, involving also other cells, such as osteocytes, bone lining cells, endothelial and immune cells, to achieve bone health [1,2]. Appropriate bone cell culture systems are widely used tools, contributing to the understanding of the cellular events and underlying mechanisms both in healthy conditions and in cases of bone disorders due to metabolic or bone defects. In this context, and targeting a translational approach, direct and indirect co-cultures of bone cells are advantageous models as they allow mimicking the interactions between the two main cells involved in bone metabolism, namely the osteoblasts and the osteoclasts, in an in vitro environment. Although these systems do not allow us to study what occurs during a remodeling cycle, where bone resorption is followed by bone formation and occurs simultaneously in different bone sites, they provide crucial information on cell-to-cell interactions and how these can affect bone remodeling [3,4]. In indirect co-cultures, the two cell types are physically separated but can be cultured under the same conditions, allowing paracrine signalling and the characterization of both cell types without the need for cell labelling [5,6]. Recurring in the use of primary or immortalized cells, these systems improve the understanding of the remodeling process, ultimately resulting in a reduction of animal testing [4,7]. Mainly, studies have focused on bone formation, less on bone resorption and scarcely on the two cell types interactions [3].

In conditions of defective bone regeneration, the use of nanomaterials is an essential promising approach, and their cytocompatibility testing in appropriate cell culture models is required before moving to more complex in vivo models. Nanomaterials used to stimulate the bone regenerative events need to be biocompatible, osteoinductive and with adequate physicochemical properties to improve bone formation while controlling bone resorption and achieving a proper equilibrium between these two processes [8,9,10]. Magnesium is the second most abundant cation in bone and is essential for bone health. Its use to synthesize nanomaterials for bone applications has shown great potential in many forms, such as filling materials or by being incorporated in scaffolds or alloys, due to its biodegradability [11]. Besides the synthesis of biomaterials being economical, the process itself needs to be more environmentally friendly, which is easily achieved through the use of medicinal plants. As such, green synthesis of magnesium oxide nanoparticles has been reported in several studies and was demonstrated to be less toxic and easier than conventional chemical synthesis [12], yielding particles with great potential biomedical applications [13,14,15,16], providing an opportunity to evolve bionanotechnology. On the other hand, biological applications of magnesium hydroxide (Mg(OH)_2_) nanoparticles have been less investigated, and green synthesis has barely been reported. Rose hip (RH), the accessory fruit of *Rosa canina* L. (the part of the flower just below the petals that contains the seeds), is a medicinal plant widely used due to its bioactive compounds, namely, polyphenols, vitamins and carotenoids, and its anti-inflammatory effects, being used as a therapeutic agent in conditions such as rheumatoid arthritis, bacterial infections and osteoporosis [17]. Due to its antioxidant properties, rose hip can play a role in restoring bone cell metabolism through the regulation of oxidative stress in cases of abnormally high levels of oxidative stress, reducing bone resorption and increasing bone formation [17,18]. We have previously reported that green-synthesized magnesium hydroxide (Mg(OH)_2_) nanoparticles, using nitrate as the magnesium counterions and rose hip extract, had affected monocultured bone cells, namely by stimulating bone-forming osteoblasts and restraining bone-resorbing osteoclasts [19]. Magnesium counterions are reported to affect crystallites and particle sizes [20]. Although subtle, these changes may lead to nanoparticles with very different physicochemical properties differentially affecting the response of biological systems [21,22].

As the effects of different Mg(OH)_2_ nanoparticles on bone have been scarcely investigated, the aim of the present work was to characterize rose hip functionalized magnesium hydroxide nanoparticles, using chloride as the counterion, on bone cells. A step forward was to address the effect of the particles on co-cultured osteoblastic and osteoclastic cells. The results were compared to those from a similar protocol set up in monocultured cells. With the more representative co-culture system, it is expected to bring about an integrative view of the biological profile of Mg(OH)_2_ nanoparticles in interacting bone cells, as well as the eventual added biological performance of the green-functionalized process as an efficient and environmentally friendly regenerative approach.

## 2. Results

### 2.1. Physicochemical Characterization of Mg(OH)_2_ Nanoparticles

**Figure 1:**
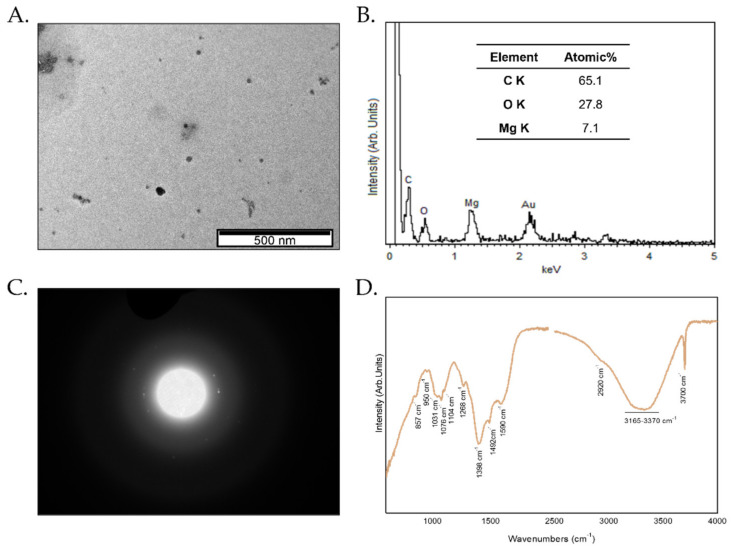
Physicochemical characterization of the Mg(OH)_2_ nanoparticles functionalized with RH. (**A**) Transmission electron microscopy (TEM) image; (**B**) energy-dispersive X-ray spectroscopy (EDS) spectrum and corresponding quantification; (**C**) selected area electron diffraction (SAED) pattern; and (**D**) attenuated total reflection Fourier-transform infrared spectroscopy (ATR FTIR) spectrum.

The size of Mg(OH)_2_RH NPs was assessed by TEM and shown in Figure 1A. These NPs present a round shape morphology with an average diameter below 10 nm. When compared with the nanoparticles synthesized without RH extract, with mean diameters of 90 nm [19], the Mg(OH)_2_RH NPs are almost 10 times smaller. The presence of Mg on the as-synthesized nanoparticles was confirmed by EDS (Figure 1B). The detection of C is related to the RH phytochemicals that are functionalizing these green-synthesized nanoparticles, and the presence of O can be correlated with both the formation of Mg(OH)_2_ and with the presence of the bioactive compounds (Figure 1B). When analyzing the SAED pattern, slightly light dots together with a blurred signal show that we are in the presence of predominantly amorphous particles (Figure 1C). These Mg(OH)_2_RH NPs, due to their small size, possess very high surface energy, which leads the NPs to aggregate in order to lower their surface energy during crystal growth. This agglomeration is reduced when the NPs were in suspension due to the presence of phytochemical in NPs’ surface. Further evidence from the phytochemical adsorbed on the surface of Mg(OH)_2_RH NPs can be obtained by the ATR-FTIR spectrum. In Figure 1D, bands at 1031 cm^−1^, 1076 cm^−1^, 1268 cm^−1^ and 1492 cm^−1^ have been assigned to polyphenols derived from RH extract. The sharp and strong peak at 3700 cm^−1^ and two small bands at 1590 cm^−1^ and 1398 cm^−1^ are due to the O–H stretching vibrations in the Mg(OH)_2_ nanoparticles structures (Figure 1D). The amount of polyphenols determined by UV-Vis present in Mg(OH)_2_RH NPs was 116 mg of tannic acid/mg particles.

### 2.2. Effect of Mg(OH)_2_ NPs in Monocultured Osteoblastic and Osteoclastic Behavior

First, the effect of Mg(OH)_2_ NPs and Mg(OH)_2_RH NPs was evaluated in monocultured osteoblastic cells (MG-63 cells) and osteoclastic cells (THP-1-derived cells), exposed to the NPs for periods up to 6 days, and cultures were characterized for viability and phenotype markers. Results were compared with those from negative control (cells grown in base medium) and positive control (cells supplemented with phenotype inducers).

#### 2.2.1. Osteoblastic Cell Behavior

**Figure 2:**
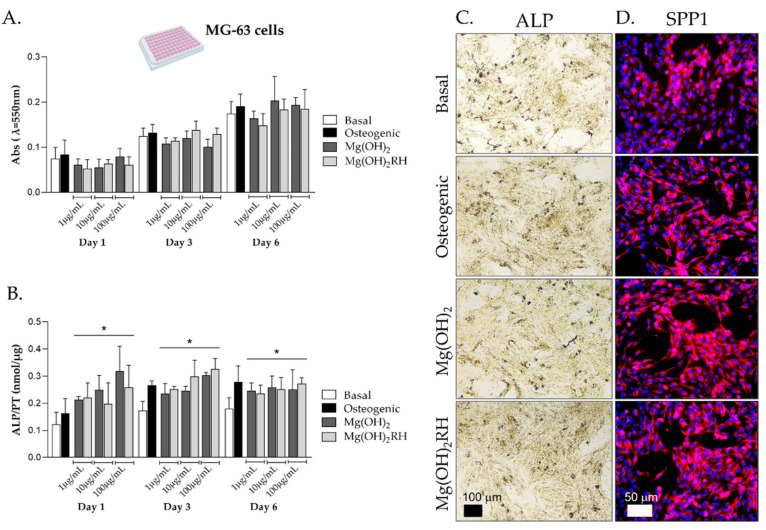
Behavior of MG-63 cells monocultured in basal and osteogenic conditions, and exposed to Mg(OH)_2_ or Mg(OH)_2_RH NPs, for periods up to 6 days. (**A**) Cell viability (MTT assay); (**B**) ALP activity, * significantly different from cultures grown in basal medium (*p* ≤ 0.05); (**C**) histochemical staining of ALP and (**D**) immunohistochemical staining of SPP1 (osteopontin) in cultures exposed to 10 μg/mL NPs for 1 day. Bar: 100 µm (**C**) and 50 µm (**D**).

MG-63 cells were cultured in basal conditions, osteogenic medium (supplementation with ascorbic acid and dexamethasone) and exposed to the NPs (1, 10 and 100 µg/mL). Cell behavior was analyzed at days 1, 3 and 6 for metabolic activity, ALP activity and staining and immunostaining of SPP1 (osteopontin) (Figure 2).

In all culture conditions, metabolic activity increased through the culture time, and no significant differences were found in the cell behavior (Figure 2A). 

ALP activity (Figure 2B) increased from day 1 to day 3 and stabilized afterwards in all conditions. Compared to basal conditions (negative control), cells cultured in osteogenic medium showed higher values on days 3 and 6. On day 1, both particles caused a dose-dependent induction in ALP activity. Additionally, the enzyme activity was higher than that measured in base medium (*p* ≤ 0.05) throughout the culture time, being similar to that observed in osteogenic conditions. No significant differences were found between the two particles. These results were corroborated through the histochemical staining of ALP, as shown in Figure 2C for cultures exposed to the NPs (10 µg/mL) for 1 day. Cultures proliferated forming cellular agglomerates that stained dark brown for ALP. Images were suggestive of a higher staining in the cultures performed in osteogenic conditions and in the presence of the NPs. A comparable pattern was noticed for the immunostaining of SPP1 (Figure 2D) that was clearly increased in the positive control and in the presence of the two NPs.

#### 2.2.2. Osteoclastic Cell Behavior

**Figure 3:**
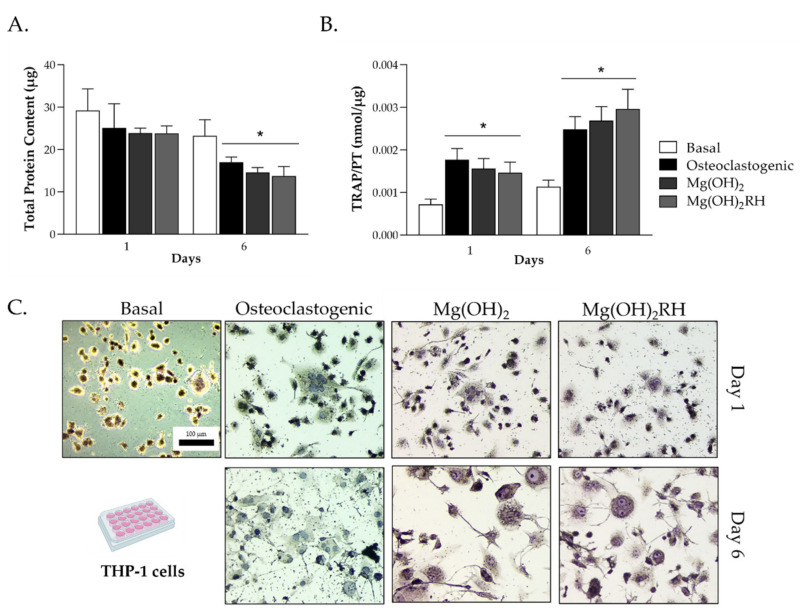
Behavior of THP-1-derived cells cultured in basal conditions, osteoclastogenic medium (supplementation with M-CSF and RANKL), and exposed to Mg(OH)_2_ and Mg(OH)_2_RH NPs (10 µg/mL) for 1 and 6 days. (**A**) Total protein content and (**B**) TRAP activity; * significantly different from the cultures grown in the basal medium (*p* ≤ 0.05); (**C**) TRAP histochemical staining of cells at days 1 and 6 in the tested experimental conditions; bar: 100 µm.

The response of THP-1-derived cells was analyzed in basal medium, osteoclastogenic conditions (presence of M-CSF and RANKL) and exposed to Mg(OH)_2_ and Mg(OH)_2_RH NPs (10 µg/mL), at days 1 and 6. In osteoclastogenic conditions and exposure to the NPs, total protein content (Figure 3A) decreased with the culture time, and values were similar in the three conditions. TRAP activity (Figure 3B) was very low in basal conditions but increased significantly (*p* ≤ 0.05) from day 1 to day 6 in the presence of the osteoclastogenic inducers or the NPs. Again, the behavior was similar in these conditions. To sustain these results, TRAP histochemical staining was performed to detect the presence of TRAP on day 1 for all conditions and on day 6 for the induced and NP-exposed cells. The osteoclastogenic factors and the NPs induced the osteoclastogenic response, as shown by the presence of TRAP(+) multinucleated cells.

### 2.3. Effect of Mg(OH)_2_ NPs in Co-Cultured Osteoblastic and Osteoclastic Cells 

MG-63 cells were cultured in Transwell^®^ inserts for adherence and after 24 h, they were indirectly co-cultured with THP-1-derived cells (seeded on the bottom of the well) for up to 6 days. THP-1-derived cells were maintained in basal conditions (control), osteoclastogenic medium (M-CSF + RANKL, positive control) and also exposed to Mg(OH)_2_ or Mg(OH)_2_RH NPs at 10 µg/mL. On days 1 and 6, each cell population was evaluated for their characteristic phenotype markers.

#### 2.3.1. Behavior of Co-Cultured Osteoblastic Cells 

**Figure 4:**
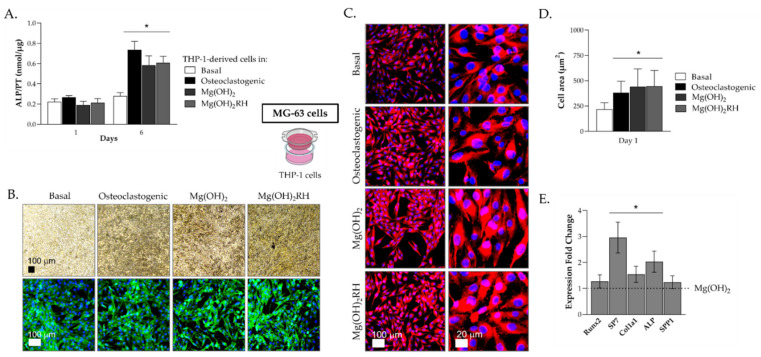
Behavior of MG-63 cells co-cultured with THP-1-derived cells. THP-1 cells were kept in basal conditions, osteoclastogenic medium (M-CSF+RANKL) and also exposed to Mg(OH)_2_ and Mg(OH)_2_RH NPs (10 μg/mL) for 1 and 6 days. (**A**) ALP activity. (**B**) Histochemical staining of ALP (upper row) and immunostaining of F-actin (green) and nucleus (blue) (lower row); bar: 100 µm. (**C**) Immunostaining of SPP1 (osteopontin) (red) and nucleus (blue); bar: 100 µm and 20 µm. (**D**) Cell area. (**E**) Expression of osteoblastic genes. (**A**,**D**): * Significantly different from cultures grown in basal medium (*p* ≤ 0.05). (**E**): * Significantly different from cultures exposed to Mg(OH)_2_ NPs (*p* ≤ 0.05).

MG-63 cells co-cultured with THP-1-derived cells were evaluated at days 1 and 6 for ALP activity and staining, immunostaining of F-actin cytoskeleton, nucleus and SPP1, and also the expression of osteogenic genes. The results are illustrated in Figure 4.

ALP activity, at day 1, was similar in all culture conditions, but values increased from day 1 to day 6. This increase was very low in MG-63 cells cultured with THP-1 cells kept in basal conditions. However, values increased significantly when THP-1 cells were cultured in osteoclastogenic conditions (~fourfold) or exposed to the NPs (~threefold), compared to day 1 (Figure 4A). The same behavior is observed in MG-63 cultures stained for alkaline phosphatase (Figure 4B, upper row). On day 6, a notorious darker staining was observed in MG-63 cultures co-cultured with THP-1 cells kept in osteoclastogenic conditions or treated with the NPs. Furthermore, cultures organized in cellular agglomerates that stained intensively for the presence of ALP. The two particles had similar behavior.

MG-63 cultures were also immunostained for the F-actin cytoskeleton and nucleus (Figure 4B, lower row). Cells presented an elongated morphology with intense cytoskeleton staining. Images suggest that cells co-cultured with THP-1 supplemented with M-CSF+RANKL or exposed to the NPs presented a higher abundance of cells showing an organized cytoskeleton surrounding the nucleus. This seems to be more notorious when THP-1 cells were exposed to the NPs, and particularly Mg(OH)_2_RH NPs.

Images of cultures immunostained for SPP1 protein (osteopontin) also suggest differences in the cell behavior (Figure 4C). MG-63 cells cultured with THP-1 cells kept in basal conditions showed a lower size and thinner morphology compared to those of the other conditions. Cells co-cultured with THP-1 cells kept with the growth factors or the NPs presented a more rounded morphology, increased cell size and intense osteopontin staining. Additionally, this was more evident when THP-1 cells were exposed to the NPs compared to the supplementation with the growth factors (Figure 4D).

The previous results were somewhat inconclusive regarding eventual differences between Mg(OH)_2_ and Mg(OH)_2_RH NPs. Therefore, MG-63 cells co-cultured with THP-1 cells exposed to the NPs were analyzed for the expression of some osteoblastic genes (Figure 4E). Overall, MG-63 cultures co-cultured with THP-1 cells treated with the green-synthesized NPs (Mg(OH)_2_RH) showed an increase in the expression of all genes compared with those co-cultured with THP-1 treated with Mg(OH)_2_ NPs. Significantly increased gene expression was observed for *ALP* (~twofold), *Col1a1* (~30%) and, particularly, *SP7* (~threefold), while the expression of *Runx2* and *SPP1* was similar in both conditions.

#### 2.3.2. Behavior of Co-Cultured Osteoclastic Cells 

**Figure 5:**
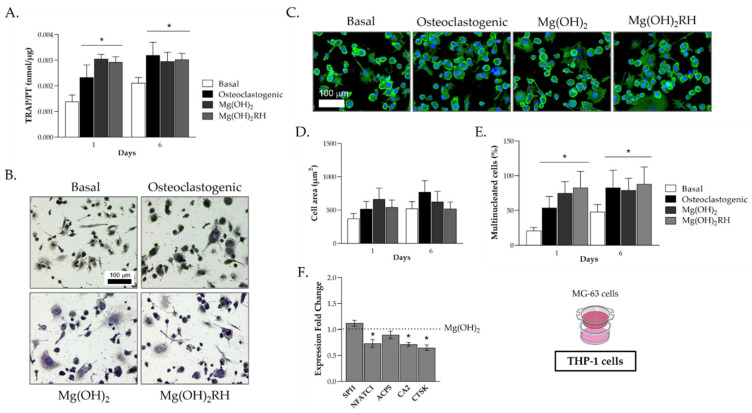
Behavior of THP-1 cells co-cultured with MG-63 osteoblastic cells for 1 and 6 days. THP-1-derived cells were cultured in basal conditions, osteoclastogenic medium or exposed to Mg(OH)_2_ or Mg(OH)_2_RH NPs (10 μg/mL). (**A**) TRAP activity; (**B**) TRAP histochemical staining, at day 6; (**C**) F-actin cytoskeleton (green) and nucleus (blue) immunostaining; (**D**) cell area; (**E**) percentage of multinucleated cells; (**F**) expression of osteoclastogenic genes. (**A**,**E**): * Significantly different from cultures grown in basal medium (*p* ≤ 0.05). (**F**): * Significantly different from cultures exposed to Mg(OH)_2_ NPs (*p* ≤ 0.05). (**B**,**C**): Bar = 100 µm.

THP-1-derived cells, kept in basal medium, osteoclastogenic conditions or exposed to the NPs, and co-cultured with MG-63 cells, were characterized for TRAP activity and staining, F-actin cytoskeleton immunostaining, cell area, percentage of multinucleated cells and expression of some osteoclastogenic genes. The results are presented in Figure 5.

TRAP activity (Figure 5A) increased from day 1 to day 6 in THP-1 cells cultured in base medium or supplemented with M-CSF and RANKL, and values were significantly higher in the induced cultures. In THP-1 cell cultures exposed to the NPs, TRAP activity peaked already at day 1 and remained similar at day 6. Enzyme activity was similar to that measured on the osteoclastogenic-induced THP-1 cells on day 6. Compared to the cells kept in basal conditions, in the other three conditions, TRAP-stained THP-1 cultures showed cells with typical osteoclastic features, namely a high number of purple-stained TRAP(+) large and multinucleated cells (Figure 5B).

THP-1-derived cells were immunostained for F-actin and nucleus at day 6 (Figure 5C) to assess cell morphology, formation of F-actin rings and evaluation of cell area and percentage of multinucleated cells for the four experimental conditions (basal, osteoclastogenic and exposure to Mg(OH)_2_ or Mg(OH)_2_RH NPs). In all conditions, cultures presented cells with osteoclastic characteristics. Compared to the basal condition, semi-quantitative evaluation of the cell area showed a tendency for an increased area in THP-1 cells induced with the growth factors or exposed to the NPs, although without attaining a statistical significance (Figure 5D). The same was not observed for the percentage of multinucleated cells (Figure 5E). An evident increase in this parameter was observed in the later conditions. Cultures exposed to Mg(OH)_2_ or Mg(OH)_2_RH NPs showed similar values at days 1 and 6 that were not statistically different from those observed in the osteoclastogenic-induced THP-1 cells.

The described evaluation of co-cultured THP-1 cells did not evidence clear differences between the two particles. However, gene expression of relevant osteoclastogenic genes (Figure 5F) revealed that cultures treated with the green-synthesized NPs showed a decrease in the expression of the genes *NFATC1*, *CA2* and *CTSK* (~25–30% reduction, *p* ≤ 0.05), compared to the Mg(OH)_2_ NP-treated cultures. Expression of *SPI1* and *ACP5* remained similar.

### 2.4. The Culture System: Monoculture vs. Co-Culture

**Figure 6:**
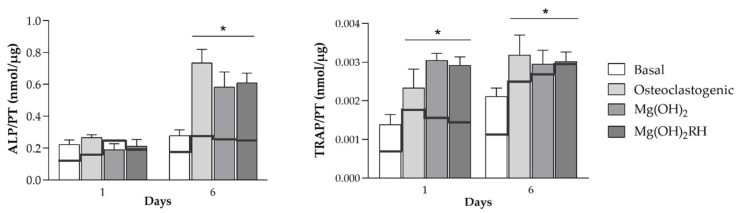
ALP activity of MG-63 cells and TRAP activity of THP-1 cells on monoculture (black continuous lines) and co-culture (bars) conditions, kept for 1 and 6 days in all tested conditions. **ALP activity**: MG-63 cells monocultured in base conditions, osteogenic medium and NPs (continuous line); MG-63 cells co-cultured with THP-1 cells, with these cells being kept in base medium, osteoclastogenic conditions and NPs (bars). **TRAP activity**: THP-1 cells monocultured in base medium, osteoclastogenic conditions and NPs (continuous line); THP-1 cells co-cultured with MG-63 cells, with THP-1 cells kept in base medium, osteoclastogenic conditions and NPs (bars). * Significantly different from the cultures grown in basal medium (*p* ≤ 0.05).

In this section, we compare the behavior of monocultured and co-cultured osteoblastic and osteoclastic cells for ALP and TRAP activities, respectively, in the tested conditions. Significant differences were noted on the two culture models (Figure 6).

Monocultured MG-63 osteoblastic cells presented low ALP activity at day 1, having a small increase at day 6 (~20%) in all conditions (basal, osteogenic, exposure to NPs). However, ALP activity increased significantly when co-cultured with THP-1 cells, particularly when these cells were kept in osteoclastogenic conditions (~fourfold) or treated with the NPs (~3 threefold), but also peaking at day 6. It should be emphasized that maximal ALP activity was significantly higher in co-cultured conditions, but attained at the same culture stage (day 6). Interestingly, in both mono- and co-cultured conditions, the effect of the NPs in ALP activity was similar to that observed in the induced conditions.

Monocultured THP-1 cells treated with the inducer factors (M-CSF and RANKL) presented high TRAP activity that increased from day 1 to day 6. Co-culturing with MG-63 cells resulted mainly in an earlier peaking of TRAP activity, i.e., maximal values were observed already at day 1, remaining similar afterwards. However, maximal TRAP activity was similar in monocultured (day 6) and co-cultured (day 1) THP-1 cells. It is also worth noting that the effect of both NPs was similar to that found in THP-1 cells cultured in osteoclastogenic conditions in both models.

## 3. Discussion

Bone regenerative medicine is one of the most complex and important field as a problem solver for imbalances and defects in the bone. Improving biomaterials aims for the needed features to reach bone regeneration such as biocompatibility, osteoinductivity, osteoconductivity and fitting mechanical and physicochemical properties [23]. The main goal is to stimulate osteoblasts’ proliferation and differentiation to achieve bone formation while regulating osteoclasts’ differentiation and promoting a balanced bone metabolism and health [9]. Nanomaterials are widely used due to their physical and chemical features, and magnesium-containing biomaterials have been associated with enhanced biocompatibility in regenerative applications. Mg plays a major role in bone metabolism and it has been reported to improve osteoblastic adhesion, proliferation [24] and differentiation through its use as a nanomaterial [19,25]. Medicinal plants are being used to direct the synthesis of NPs to be more ecologic, further allowing their functionalization with biological properties such as antioxidant, anti-inflammatory and/or antibacterial activity [13,14,19,26,27,28].

This work focused on the effect of green-synthesized Mg(OH)_2_ nanoparticles in bone cells recurring to an indirect co-culture system of human osteoblasts and osteoclasts. The aim was to better characterize the integrative response of the NPs on interacting osteoblastic and osteoclastic cells and, further, to assess the potential of green nanoparticles for bone regenerative applications. For that, Mg(OH)_2_RH NPs synthesized through chloride precursors were compared to a control (Mg(OH)_2_) where the synthesis occurred in pure water [19]. In a previous work [19], we reported the synthesis of these NPs, but using a different precursor, nitrate, as the magnesium counterion, as it was described that the precursors may have an important role in the characteristics of produced Mg(OH)_2_ nanoparticles [29]. However, when comparing the nanoparticles synthesized herein using chlorides with the nanoparticles synthesized with nitrates [19], no significant differences were observed in size, morphology or RH phytochemicals loading, suggesting that the rose hip extract has a preponderant role in the final characteristics of the NPs and not the precursor. Nevertheless, polyphenol release kinetics were not evaluated for the two particles, and small differences might affect differently the diverse biological profile associated with polyphenols, namely antioxidant and anti-inflammatory effects and antibacterial activity [30]. This is now under investigation by our group. 

The first in vitro testing approach was to analyze the effect of the two types of Mg(OH)_2_ NPs in monocultured osteoblastic and osteoclastic cells. Human cell lines were used as an option for primary cells, namely the MG-63 osteoblastic cell line [31,32] and the osteoclastic-differentiated THP-1 cells [33]. The use of cell lines as an alternative to primary cells is widely accepted due to the difficulties faced by using primary cells. The main concern is the patient-to-patient variability, which is minimized using cell lines, translating into higher phenotypic stability and allowing for greater reproducibility and more reliable comparison of different studies [31,34,35].

MG-63 is a proliferative osteoblastic cell line, characterized for its pre-osteoblast stage and having some important phenotypical similarities to human primary osteoblasts, namely hormonal response and integrin subunits profile and sensitivity to osteogenic differentiation inducers [32] being widely used to test biomaterials [31,34]. In order to verify the suitability of MG-63 cells as an osteoblastic cell model, these cells were monocultured in basal and osteogenic medium (supplementation with ascorbic acid and dexamethasone [19]). Results showed increased ALP activity and staining in osteogenic conditions (Figure 2B,C), supporting the use of these cells as negative (basal conditions) and positive (osteogenic medium) controls. Monocultured MG-63 cells were also sensitive to the exposure to Mg(OH)_2_ NPs, reflected by an induction in ALP activity, with maximal values similar to that found in osteogenic conditions (Figure 2B). This positive effect is in line with previous studies involving Mg-containing NPs and other materials [19,36,37,38]. The inductive effect of the NPs occurred soon after the exposure, observed already at day 1, which did not occur by culturing the cells in osteogenic medium with a delayed induction (days 3 and 6). 

The effect of Mg(OH)_2_ NPs was also assessed in osteoclastic cells, by using a human osteoclast model established from the differentiation of the monocytic cell line THP-1 [33]. THP-1 cells were first differentiated into macrophage-like cells with PMA and then induced to osteoclastic cells with M-CSF and RANKL [33,39]. The differentiation of macrophages into osteoclasts was verified by analyzing TRAP activity and the formation of TRAP(+) multinucleated cells, which were all greatly increased in the induced cells, as expected for the osteoclastic differentiation [40]. As such, cultures performed in basal conditions or induced with the growth factors were used as negative and positive controls, respectively. THP-1-derived cells were also highly sensitive to Mg(OH)_2_ NPs. The two types of particles were able to induce the osteoclastic features to levels similar to those observed with the induction factors (M-CSF and RANKL), following a similar pattern, results that are in line with previous work with similar NPs [19].

The results mentioned above suggest that both the MG-63 cell line and THP-1-derived cells are sensitive to the effects of Mg(OH)_2_ and Mg(OH)_2_RH NPs. Following, the cell response to these NPs (10 µg/mL) was analyzed in an indirect co-culture model of these cell types, an approach that has not yet been addressed before. This model allows reciprocal cellular communication, providing a more representative and relevant system to test the NPs for bone applications [4,5,7,41]. In the present study, Transwell^®^ inserts with MG-63 cells previously cultured for 24 h were fitted on top of the 24-well plates with the cultured THP-1-derived cells. THP-1 cells were kept in four experimental conditions, i.e., base medium (negative control), osteoclastogenic conditions (M-CSF and RANKL, positive control) and exposed to Mg(OH)_2_ or Mg(OH)_2_RH NPs. Co-cultured cells were analyzed individually for the respective phenotype parameters.

**Figure 7:**
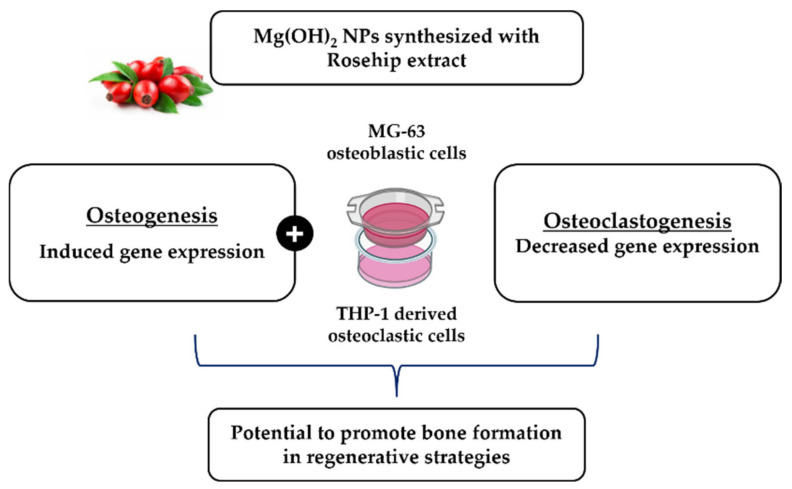
Potential added value of green-synthesized NPs, Mg(OH)_2_RH NPs, to promote osteogenesis in bone regenerative strategies.

The results showed that co-cultured MG-63 cells were very sensitive to the culture conditions of interacting THP-1 cells (Figure 4). Culture of THP-1 cells in osteoclastogenic conditions or in the presence of the NPs had a significant effect on the behavior of MG-63 cells, evidenced by a fourfold or a threefold increase in ALP activity, respectively, at day 6. This induction was also evident in the immunohistochemical staining of osteopontin. The co-culture conditions provide essential paracrine interactions needed for cell activity [42,43]. As mentioned above, the positive effects of Mg-containing NPs in osteoblastic cells have been reported both in vitro and in vivo conditions [11,19,24,25]. However, the present study reports for the first time that the inductive osteoblastic effect increases significantly in interacting osteoblastic and osteoclastic cells, a condition that better mimics the bone cellular environment. Nevertheless, the above positive effects of Mg(OH)_2_ NPs in the osteoblastic behavior, did not allow us to detect differences between the conventional or the green-synthesized NPs. This aspect was elucidated at the molecular level by analyzing the gene expression of relevant osteoblastic markers in MG-63 cells co-cultured with THP-1 cells exposed to Mg(OH)_2_ or Mg(OH)_2_RH NPs. Expression of early osteogenic differentiation markers such as *Runx2*, *Collagen type I* and *ALP*, and also later differentiation markers such as *Osterix* (*SP7*) and *Osteopontin* (*SPP1*) were higher in cultures exposed to the green-synthesized NPs. Significant upregulation was observed for the genes coding for collagen type I (~30%), the main component of the bone matrix, ALP (~twofold), an enzyme needed for the initiation of the matrix mineralization [44] and, particularly, osterix (~threefold), a later transcription factor essential for osteoblast differentiation and bone formation [44]. This indicates an added osteogenic differentiation potential by the presence of the rose hip extract (Figure 7), most probably related with its polyphenolic content. The involvement of polyphenols, such as flavonoids, in tissue regeneration has already been reported in studies related to oral and bone applications [17,38,45,46,47,48].

Osteoclastic response in bone interacting cells is also relevant in the regeneration process. Co-cultured THP-1-derived cells were also evaluated for osteoclastic behavior (Figure 5). On THP-1 cells supplemented with the induction factors (M-CSF and RANKL) or exposed to the NPs, TRAP activity attained maximal values already at day 1, and values remained similar at day 6. There were no significant differences in these situations. The same was observed for the cell area and the percentage of multinucleated cells. The results showed that in interacting osteoclastic and osteoblastic cells, the two NPs elicited a response similar to that induced by the osteoclastogenic factors, following a similar temporal pattern.

Differences between Mg(OH)_2_ or Mg(OH)_2_RH NPs were disclosed only at the gene expression level. The green-synthesized NPs caused a decrease in the expression of *NFATC1*, *CA2* and *CTSK*. *NFATC1* is a key transcription factor for osteoclastogenesis given its key role as an inducer for osteoclastic gene markers expression such as *ACP5* (also known as *TRAP*) and *CTSK*, and also being involved in the differentiation of monocytes and macrophages into osteoclasts [49,50]. *CA2* and *CTSK* are associated with bone resorption and are normally expressed by resorbing osteoclasts, being major indicators of osteoclastogenesis [51]. These findings suggest an eventual potential to decrease osteoclastogenesis and osteoclastic function of the Mg green-synthesized NPs, which is aligned with the presence of polyphenols in the green extract, agreeing with previous studies performed in mouse bone marrow cells [52]. The effect of these compounds in the activation of enzymes that diminish inflammatory processes leads to the impairment of osteoclast differentiation [48].

Although the main focus of this work was to analyze the effect of Mg(OH)_2_ NPs in interacting osteoblastic and osteoclastic cells, it is worth noting that the cell behavior was greatly affected by the culture conditions, i.e., monoculture versus co-culture, as it is already well established, including in studies using a magnesium extract [43]. In the present work, the effects were noticed in the two interacting cell populations. In the osteoblastic cells, the main effect was a significantly higher induction of ALP activity in the co-culture with THP-1-derived cells. On the other hand, a much earlier production of TRAP by THP-1 cells was observed when interacting with the osteoblastic cells, although maximal attained TRAP activity was similar in both mono- and co-culture conditions. The complex paracrine interactions between the two cell types will depend on a multicity of factors, such as the specific cell lines, culture conditions and stage of cell differentiation, and the present results do not allow us to explain the observed differences. Nevertheless, it is interesting to note that in both mono- and co-cultured conditions, the effect of the NPs in ALP activity or TRAP activity followed a pattern similar to that observed in the induced phenotype conditions for each cell population.

## 4. Materials and Methods

### 4.1. Synthesis and Physicochemical Characterization of Mg(OH)_2_ Nanoparticles

Mg(OH)_2_ NPs were synthesized as described elsewhere [19], but by using magnesium chloride (MgCl_2_·6H_2_O, Sigma-Aldrich, St. Louis, MI, USA) as the precursor. Briefly, the process was carried out in pure water or recurring to a green synthesis process by using a 75% aqueous rosehip (RH) extract. Two NPs were obtained, Mg(OH)_2_ and Mg(OH)_2_RH.

To produce the RH extract, 10 g of dried RH berries were added to 500 mL of distilled water and boiled for about 45 min. After this time, the suspension was filtered with MACHEREY-NAGEL paper filters to remove the RH berries. The extract obtained was subsequently used to produce the NPs. In preliminary experiments, NPs were prepared using different concentrations of the extract (25%, 50%, 75%, results not shown), and the NPs produced with 75% extract had the highest amount of phytochemicals per mg of nanoparticles. These NPs are expected to elicit the most effective and efficient biological response, and, for this reason, they were selected to be prepared in this study.

Characterization of the size and shape of NPs was assessed by transmission electron microscopy (TEM) using a Hitachi H-9000-NA microscope operating at 200 kV with supporting copper–carbon grids. The chemical characterization of Mg(OH)_2_RH NPs was achieved with Fourier transformed infrared spectroscopy (FTIR) using a Nicolet (Thermo Electron) spectrometer with an attenuated total reflectance (ATR) apparatus. The total phenolic content of the nanoparticles was e evaluated by the Folin–Ciocalteu method [53] and is expressed in mg tannic acid equivalents/mL.

### 4.2. Cell Cultures 

#### 4.2.1. MG-63 Cell Monocultures and Exposure to Mg(OH)_2_ NPs

MG-63 (ATCC^®^CRL-1427™) cells were cultured in 96-well plates (2 × 10^4^ cells/cm^2^) in basal medium containing RPMI-1640 medium supplemented with 10% fetal bovine serum (FBS), 100 IU/mL penicillin, 100 µg/mL streptomycin and 2.5 µg/mL amphotericin B (basal medium; all reagents from Gibco) at 37 °C, 95% humidity and 5% CO_2_ atmosphere. After a 24 h incubation period for adherence, the medium was substituted for fresh basal medium or basal medium supplemented with 50 µg/mL ascorbic acid and 10 nM dexamethasone (osteogenic medium; all reagents from Sigma-Aldrich), used as controls, or exposed to Mg(OH)_2_ NPs (1, 10 and 100 µg/mL). This concentration range was selected based on a previous work performed with similar NPs, showing its cytocompatibility in these levels [19]. Cultures were grown until day 6 and characterized for metabolic activity (MTT assay), alkaline phosphatase (ALP) activity and histochemical staining, and immunostaining of nucleus and osteopontin (SPP1).

#### 4.2.2. THP-1 Cell Monocultures and Exposure to Mg(OH)_2_ NPs

THP-1 (ATCC^®^TIB-202™) monocytic cells were suspended (1.25 × 10^5^ cells/cm^2^) in basal medium containing RPMI-1640 medium supplemented with 10% fetal bovine serum (FBS), 100 IU/mL penicillin, 100 µg/mL streptomycin and 2.5 µg/mL amphotericin B (All reagents from Gibco). For differentiation into macrophage-like cells, medium was supplemented with 100 ng/mL phorbol 12-myristate 13-acetate (PMA, Sigma-Aldrich), and plates were then incubated at 37 °C, 95% humidity and 5% CO_2_ atmosphere for 48 h. Osteoclastic differentiation was achieved by supplementation of the medium with 50 ng/mL of Macrophage colony-stimulating factor (M-CSF) and 50 ng/mL of receptor activator of nuclear factor kappa-B ligand (RANKL) (osteoclastogenic medium; both from PeproTech) or exposed to Mg(OH)_2_ NPs (10 µg/mL). Cell response was evaluated at days 1 and 6 after osteoclastic differentiation for total protein content, tartrate-resistant acid phosphatase (TRAP) activity and histochemical staining.

#### 4.2.3. Indirect Co-Cultures of MG-63 Osteoblastic Cells and THP-1-Derived Macrophages and Exposure to Mg(OH)_2_ NPs

THP-1 cells were cultured in 24-well plates as before and differentiated into macrophage-like cells. After, Transwell^®^ inserts (0.33 cm^2^, 0.4 µm polyester membrane) with MG-63 cells previously cultured for 24 h in osteogenic medium were fitted on top of the 24-well plates with the cultured THP-1-derived cells. Subsequently, THP-1-derived cells medium was exchanged by basal medium or osteoclastogenic medium, and MG-63 cells were cultured in osteogenic medium. Both cell types were exposed to Mg(OH)_2_ NPs (10 µg/mL). Cell response was characterized for each cell type at days 1 and 6 after exposure. MG-63 cells were evaluated for alkaline phosphatase (ALP) activity and staining, immunostaining of F-actin cytoskeleton, nucleus and osteopontin (SPP1) and gene expression of osteoblastic markers. THP-1-derived cells were characterized for tartrate-resistant acid phosphatase (TRAP) activity and staining, immunostaining of F-actin cytoskeleton and nucleus, cell area, percentage of multinucleated cells and gene expression of osteoclastic markers.

### 4.3. Cell Characterization

#### 4.3.1. Metabolic Activity (MTT Assay)

Metabolic activity was assessed in MG-63 cell monocultures through the MTT assay on days 1, 3 and 6. MTT (5 mg/mL, Sigma-Aldrich) was added, and the cultures were incubated for 3 h at 37 °C. Then, culture medium was removed, and dimethyl sulfoxide (DMSO, Panreac) was added (room temperature, 15 min) to dissolve the formazan salts. Absorbance was measured at λ = 550 nm in a microplate reader (Synergy HT, Biotek).

#### 4.3.2. Alkaline Phosphatase Activity and Staining

The ALP activity of MG-63 cell monocultures and co-cultures was evaluated on days 1, 3 and 6 in cell lysates (Triton X-100 0.1%, 30 min), by the hydrolysis of p-nitrophenyl phosphate (p-NPP, 25 mM, Sigma-Aldrich) in an alkaline buffer (pH 10.3, 37 °C, 1 h). The reaction was stopped with NaOH 5 M, and the product (p-nitrophenol) was measured at λ = 400 nm in a microplate reader (Synergy HT, Biotek). Results were normalized to total protein content and expressed as nanomoles of p-nitrophenol per microgram of protein (nmol/µg protein).

For ALP staining, MG-63 mono and co-cultures were fixed in glutaraldehyde 1.5% (TAAB) in sodium cacodylate buffer 0.14 M (Sigma-Aldrich) for 15 min. Fixed cultures were incubated in a filtered solution containing sodium naphthyl phosphate (2 mg/mL, Sigma-Aldrich) and Fast Blue RR in Tris buffer solution 0.1 M, pH 10 (2 mg/mL, Sigma-Aldrich) for 1 h, protected from light. Stained cultures were observed by light microscopy (Primo Vert™ Inverted Microscope, Carl Zeiss). ALP presents a brown to black staining.

#### 4.3.3. Total Protein Content

Total protein content was quantified in both mono and co-cultures of MG-63 cells on days 1, 3 and 6 and THP-1-derived cells on days 1 and 6. Cell lysates (Triton X-100 0.1%, 30 min) were obtained and then evaluated using the DCTM Protein Assay (BioRad), according to the manufacturer’s instructions. 

#### 4.3.4. Tartrate-Resistant Acid Phosphatase Activity and Staining

TRAP activity was evaluated on days 1 and 6. Cell lysates of THP-1-derived cell cultures (Triton X-100, 30 min) were evaluated by the hydrolysis of p-nitrophenyl phosphate 25 mM (p-NPP) in tartaric acid buffer (0.04 M tartaric acid and 0.09 M citrate, pH 4.8), at 37 °C for 1 h. The reaction was stopped with NaOH 5 M, and absorbance was measured at λ = 400 nm in a microplate reader (Synergy HT, Biotek). Results were normalized to total protein content and expressed as nanomoles of p-nitrophenol per microgram of protein (nmol/µg protein).

TRAP staining was assessed on days 1 and 6 in cultures fixed for 10 min with formaldehyde 3.7% (Sigma-Aldrich) and stained using the Leukocyte Acid Phosphatase (TRAP) kit (Sigma-Aldrich) according to the manufacturer’s instructions. Stained cultures were evaluated in a Primo Vert™ Inverted Microscope for the presence of TRAP(+) cells, stained purple. 

#### 4.3.5. Immunostaining of SPP1 Protein, F-Actin Cytoskeleton and Nucleus

MG-63 cells in co-culture exposed to Mg(OH)_2_ NPs (10 µg/mL) were fixed (formaldehyde 3.7%, 10 min), permeabilized (Triton X-100 in PBS, 0.1%, 30 min, room temperature) and incubated with bovine serum albumin (BSA in PBS, 1%, 30 min, Sigma-Aldrich). Cultures were incubated with the primary antibody, Purified anti-Osteopontin (SPP1) Antibody (2.5 µg/mL, overnight, BioLegend) and then incubated with the secondary antibody, Alexa Fluor^®^ 594 Goat anti-mouse IgG (minimal x-reactivity) Antibody (5 µg/mL, 2 h, BioLegend). For F-actin cytoskeleton and nucleus staining, cells were incubated with Alexa Fluor^®^ 488 phalloidin (1:100, 30 min, Molecular Probes) and then Hoechst (8 µg/mL, 10 min, Enzo). Images were obtained using the Celena S digital imaging system (Logos Biosystems).

THP-1-derived cells in co-culture exposed to Mg(OH)_2_ NPs (10 µg/mL) were fixed (formaldehyde 3.7%, 10 min), permeabilized (Triton X-100 in PBS, 0.1%, 15 min, room temperature) and incubated with bovine serum albumin (BSA in PBS, 1%, Sigma-Aldrich) to reduce non-specific coloring. Cultures were stained for F-actin cytoskeleton with Alexa Fluor^®^ 488 phalloidin (1:100, 30 min, Molecular Probes), and nucleus with Hoechst (8 µg/mL, 15 min, Enzo). Images were obtained using the Celena S digital imaging system (Logos Biosystems). Cell area was evaluated using the measure tool, and the percentage number of multinucleated cells (≥3 nuclei) was calculated in the ImageJ software v.1.53f.

#### 4.3.6. Real-Time Quantitative Polymerase Chain Reaction (RT-qPCR)

Osteogenic differentiation of MG-63 cells and the osteoclastogenic differentiation of THP-1 cells in co-culture, both cell types exposed to Mg(OH)_2_ NPs (10 µg/mL), were assessed on day 1 by real-time quantitative polymerase chain reaction (RT-qPCR). Total RNA was extracted using the TRIzol™ reagent (Invitrogen) and reverse-transcribed into complementary DNA (cDNA) with the NZY First-Strand cDNA Synthesis Kit (Nzytech), according to the manufacturer’s instructions. The expression of the target genes (Table 1) was quantitatively determined on RT-PCR equipment (CFX96, Bio-Rad) using iQTM SYBR^®^ Green Supermix (BioRad). 

### 4.4. Statistical Analysis

All data were obtained from three separate experiments, each one performed in triplicate, and expressed as mean values ± standard deviation. Statistical analysis was performed using the IBM^®^ SPSS^®^ Statistics 25. Comparison of experimental conditions was assessed using the *t*-test and the groups were compared by the one-way analysis of variance (ANOVA), followed by the post hoc Tukey test. For both, *p*-values ≤ 0.05 were considered significant.

## 5. Conclusions

Magnesium hydroxide NPs produced by a classical chemical process (Mg(OH)_2_ NPs), or green-synthesized using a rose hip extract (Mg(OH)_2_RH NPs) were evaluated in an indirect co-culture system of osteoblastic and osteoclastic cells, which allowed reciprocal paracrine interactions between the two cell types. Both particles greatly induced ALP activity, but also increased TRAP activity in a way similar to that observed in the presence of osteoblastic and osteoclastic inducers, suggesting an increase in bone remodeling dynamics. Differences between Mg(OH)_2_ NPs and Mg(OH)_2_RH NPs were evident in the gene expression profile of each cell population. The green synthesized NPs greatly increased the expression of the osteoblastic genes coding for ALP, collagen type I and osterix, and decreased the osteoclastogenic genes coding for the transcription factor NFATC1 and the functional markers CA2 and CTSK. Overall, a positive added effect could be hypothesized for the green-synthesized Mg(OH)_2_RH NPs with potential usefulness to promote bone formation in regenerative applications.

## Figures and Tables

**Table 1:** Genes and respective primers assay ID (BioRad) for RT-qPCR.

Gene	Gene Name	Assay ID
Reference	Glyceraldehyde-3-Phosphate Dehydrogenase (GADPH)	qHsaCED0038674
Osteoblastic	Runt-related transcription factor 2 (Runx2)	qHsaCED0044067
	SP7 transcription factor (SP7)	qHsaCED0003759
	Collagen type I alpha I chain (Col1α1)	qHsaCED0043248
	Alkaline phosphatase (ALP)	qHsaCED0045991
	Secreted Protein Acidic and Rich in Cysteine (SPARC), aka Osteonectin	qHsaCID0010332
	Tumor Necrosis Factor Receptor Superfamily Member 11b (TNFRSF11B), aka Osteoprotegerin	qHsaCED0046251
Osteoclastic	Spi-1 proto-oncogene (SPI1)	qHsaCID0022097
	Nuclear factor of activated T cells 1 (NFATC1)	qHsaCED0044370
	Acid phosphatase 5, tartrate-resistant (ACP5)	qHsaCED0056724
	Carbonic anhydrase II (CA2)	qHsaCID0021039
	Cathepsin K (CTSK)	qHsaCID0016934

## Data Availability

The data presented in this study are available on request from the corresponding author.
